# Female adipose tissue has improved adaptability and metabolic health compared to males in aged obesity

**DOI:** 10.18632/aging.102709

**Published:** 2020-01-26

**Authors:** Varghese Mita, Griffin Cameron, McKernan Kaitlin, Eter Leila, Abrishami Simin, Kanakadurga Singer

**Affiliations:** 1 Department of Pediatrics, Division of Endocrinology, University of Michigan Medical School, Ann Arbor, MI 48109, USA

**Keywords:** adipose tissue macrophages, aging, senescence, extracellular matrix remodeling, sex differences

## Abstract

Aging, like obesity, is associated with metabolic and inflammatory alterations within adipose tissue in older individuals. Younger females are protected from adipose inflammation, but older post-menopausal females exhibit exaggerated visceral adiposity correlated with increased disease risk. Obesity accelerates the onset and progression of age-associated diseases, but it is unclear if aging and obesity drive adipose tissue dysfunction in a sexually dimorphic fashion. We investigated adipose tissue metabolism and inflammation in a diet-induced obesity model in young and old mice. We identified age related sex differences in adipose tissue macrophages (ATMs), fibrosis and lipid metabolism in male and female visceral fat depot (GWAT). Although aging normalized body weights between the sexes, females remained protected from proinflammatory ATMs and stimulated lipolysis failed to adversely affect the inflammatory state even with obesity. Older obese males had augmented CD11c^+^ ATMs and higher insulin levels, while females showed increased visceral adiposity and exaggerated *Pparγ,* and *Pgc1α* expression. Obesity in aging demonstrated similar expression of GWAT *p53, p16, p21, Timp1* and *Tgfβ1* in both sexes. Our studies suggest that even with aging, female GWAT shows an attenuated inflammatory response compared to males due to an efficient oxidative metabolism combined with an active tissue remodeling state.

## INTRODUCTION

Aging is associated with an increased risk for cardiovascular diseases, especially in older men and women [1, 2]. Studies have shown that while young female mice have smaller infarcts after an experimental stroke compared to males, this phenotype is reversed in older animals [3]. The underlying mechanism behind this changed phenotype in aged females is unknown. One potential factor may be the age-related increase in adipose tissue in women during menopause, leading to increased adipose tissue inflammation and an enhanced systemic pro-inflammatory environment prior to the stroke. Obesity is a primary cause for this adipose tissue expansion and is a well-known predictor of cardiovascular disease in both sexes. While some cardiovascular risk factors, such as heart disease, are more prevalent in younger men, the menopausal transition in women is associated with a significant increase in body weight and abdominal fat in older females [4].

Adipose tissue, while being a primary energy storage site, is now recognized as an endocrine organ that plays a key role in obesity-induced systemic inflammation. Adipocyte hypertrophy is an adaptive response to nutrient excess that maintains adipose tissue nutrient buffering capacity and protects other tissues from lipotoxicity. In obesity, inflammation is initiated by the inability of adipose tissue to buffer dietary lipids resulting in lipotoxicity mediated by the ectopic deposition of lipids in other organs [5, 6]. Lipotoxicity in these tissues increases reactive oxygen species and activates serine threonine kinases such as c-jun N-terminal kinase (JNK), IκB kinase (IKK), and protein kinase C (PKC). These events disrupt insulin receptor signaling cascades and promote insulin resistance [5]. In addition, bioactive lipid metabolites, diacylglycerol and ceramides, accumulate and negatively impact mitochondrial function, and biogenesis that are associated with the development of hepatic steatosis and muscle dysfunction [7, 8]. Hence, directly linking inflammation with adipocyte dysfunction.

Obesity-induced inflammation is specifically characterized by the infiltration and retention of immune cells within the adipose tissue known as adipose tissue macrophages (ATMs). Obesity leads to a shift in the activation state of ATMs from an M2-polarized state in lean animals that may protect adipocytes from inflammation to an M1 proinflammatory state [9]. Infiltrating ATMs release pro-inflammatory cytokines and chemokines including tumor necrosis factor α (TNFα), interleukin 1β, interferon γ, monocyte chemo-attractant protein (MCP1) and interleukin 6 (IL6) [10]. This type of adipose tissue inflammation, specifically within the visceral-gonadal white adipose tissue (GWAT), has been linked to the metabolic syndrome [11, 12]. With old age, fat distribution shifts from subcutaneous to visceral fat depots, and triglycerides ectopically deposit on liver, muscle, bone marrow, and heart [13]. Obesity accelerates the onset and progression of a variety of age-associated diseases, further emphasizing the level of impact adipose tissue plays in aging [14]. Adipose tissue dysfunction in aging affects the immune system function characterized by aberrant chronic low-grade inflammatory response known as “inflammaging” [15]. In this context, the M1 and M2 type macrophage responses may be dysregulated in the aging mice. However, there is a lack of studies comparing such inflammatory responses in both sexes with aging and obesity.

Another impact of aging related to sex differences is an overall decline in levels of estrogens and progesterone in females and testosterone in males [16, 17]. Postmenopausal women are prone to be obese and more likely to develop type 2 diabetes compared to men and younger women [18, 19]. Changes in sex steroid concentrations and sex steroid receptor signaling with age and obesity may contribute to age-associated dysregulation of immune function [3, 20, 21]. Among women, with menopause, numbers of B and T cells are reduced and concentrations of IL-1β, IL-6, and TNF-α are significantly increased [22, 23]. Treatment of post-menopausal females with hormone replacement therapies that contain estrogen formulations effectively reduces baseline concentrations of proinflammatory cytokines when compared with post-menopausal females not on treatment [18, 23]. Whether testosterone replacement therapy affects immune responses in aged human males has not been reported.

Understanding the effects of aging on immune responses within the adipose tissue is important. The majority of experimental studies on inflammation have been performed using genetic- or diet-induced obesity in young male animals. This study includes young and aged mice of both sexes to investigate the effects of sex and aging on adipose tissue mass and pro-inflammatory responses. We identified age related sex differences in inflammation, fibrosis and lipid metabolism in aging male and female visceral fat depots. We also investigated whether excess free fatty acid (FFA) accumulation with stimulated adipose tissue lipolysis by a β_3_-adrenergic receptor (ADRB3) agonist led to an additional varied inflammatory response in the older females. Our studies show that older obese females were still protected from proinflammatory macrophages and stimulated lipolysis did not adversely affect the inflammatory state. However, our studies suggest that efficient oxidative metabolism in older obese female adipose tissue compared to males combined with an active tissue remodeling state lead to this attenuated inflammatory response even with aging.

## RESULTS

### Aging and high fat diet exposure promotes more visceral and subcutaneous adiposity in females but leads to hepato-steatosis in males

**Figure 1:**
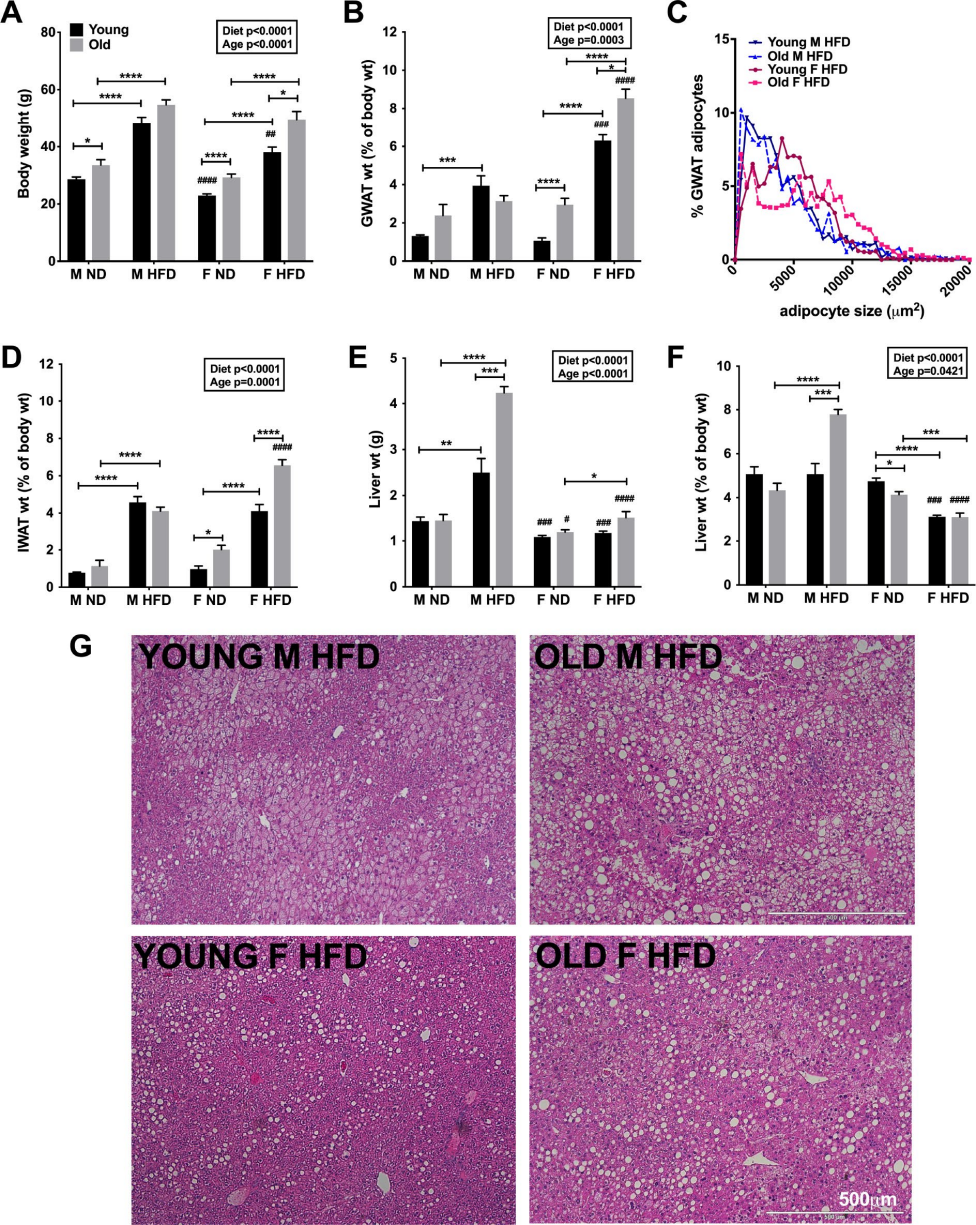
**Aging and HFD feeding induced sex differences in total body adiposity and tissue weights.** (**A**) Body weights of C57Bl6/j male and female on ND or 60% HFD starting at 6-week of age (young) or after 10 months of age (old) for 24-week. (**B**) GWAT percent weight. (**C**) Relative distribution of GWAT adipocyte cross-sectional area (**D**) IWAT percent weight. (**E**) Liver weight. (**F**) Liver percent weight. (**G**) H&E staining of liver sections depicting lipid accumulation in young and old obese male and females. Scale bar = 500 μm. N=7-12 /group. Two-way ANOVA with Bonferroni-Dunn’s post-test was performed for (**A**, **B**) and (**D**–**F**). Statistics from diet and sex interaction are in box. One-way ANOVA with Sidak’s post-test was performed for (**C**). Statistical significance is indicated by *p<0.05, **p<0.01, ***p<0.001, ****p<0.0001. Student’s t-test was performed for male and female comparisons between the same diet groups indicated by ^#^p<0.05, ^##^p<0.01 ^###^p< 0.001 and ^####^p<0.0001; error bars are SEM.

To determine differences in age induced metabolic changes, we used young (6-week) and old (10 months) mice that were placed on normal diet (ND) or high fat diet (HFD) for 24-week (see Materials and Methods). Our study included both male and female mice in order to assess any sex differences in aging and HFD responses. We then examined body adiposity, adipose tissue, and liver mass. Diet and age had significant effect on all parameters (two-way ANOVA, p<0.05; Figure 1A–1E). In both states, young and old, HFD mice were heavier than ND mice. Older animals on ND and HFD weighed more than their younger counterparts in both diet conditions (Figure 1A). Amongst the younger mice, females weighed less than the males (Figure 1A). However, in old age, females were similar in weight to males fed both ND and HFD (Figure 1A).

Both young and old female mice on HFD showed significant visceral adiposity as demonstrated by larger GWAT fat pads in grams and as a percentage compared to males (Figure 1B). Amongst the males, only the young males showed an increase in GWAT weight with HFD (Figure 1B). In young mice after 24-week of HFD, the average GWAT adipocyte size was found to be larger in females. However, aging further exacerbated the differences with older females showing relatively higher abundance of larger adipocytes in the GWAT (one-way ANOVA, p<0.05; Sidak’s post-test, *p<0.05; Figure 1C). Similar to GWAT, subcutaneous inguinal WAT (IWAT) weights increased in young and old females with HFD challenge but showed no differences by age in males (Figure 1D). However, obesity in aging female mice led to significantly increased IWAT weights compared to males (Figure 1D). Aging male mice on HFD showed significantly increased liver weights suggesting lipid accumulation in the liver (Figure 1E and 1F). In contrast, liver weights remained lower in females even with obesity in young and old animals (Figure 1E and 1F). In young age, obese male livers showed micro and macro-steatosis compared to females (Figure 1G, *top and bottom left*). Concurrently with increase in liver weights (Figure 1E and 1F), greater hepatic lipid accumulation was observed in H&E stained liver sections of older and obese males (Figure 1G, *top right*) than females (Figure 1G, *bottom right*).

These results suggest that aging led to a shift in fat accumulation in the liver in older males with obesity compared to the adipose depots, while older obese females continued to expand their adipose fat depots.

### Old and young obese females are protected equally from pro-inflammatory CD11c^+^ ATM accumulation in visceral adipose tissue

**Figure 2:**
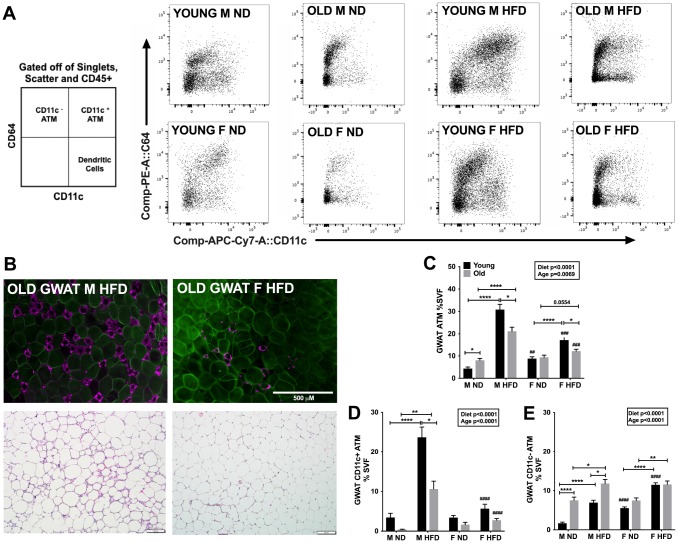
**Aging and obesity promote pro-inflammatory ATMs in young and old male mice GWAT.** (**A**) Representative flow cytometry gating strategy for CD64^+^CD11c^+^ ATMs in GWAT SVF derived from ND and HFD fed young and old mice. (**B**) *top row*- Immunofluorescence images of old male obese GWAT and old female obese GWAT depicting MAC-2 labeling of CLS (magenta) and CAV-1 labeling of adipocytes (green). Scale bar = 500 μm. (**B**) *bottom row*- H&E staining of GWAT sections depicting CLS in old obese male and females. Scale bar = 500 μm. Quantitation as a % of SVF of (**C**) GWAT ATMs (**D**) GWAT CD11c^+^ ATMs (**E**) GWAT CD11c^-^ ATMs. N=7-12/group. Two-way ANOVA with Bonferroni-Dunn’s post-test was performed for (**C**, **E**). Statistics from diet and sex interaction are in box. Statistical significance is indicated by *p<0.05, **p<0.01, ***p<0.001, ****p<0.0001. Student’s t-test was performed for male and female comparisons between the same diet groups indicated by ^#^p<0.05, ^##^p<0.01 ^###^p<0.001 and ^####^p<0.0001; error bars are SEM.

In rodent models of obesity, macrophage accumulation depicted by macrophage clustering around adipocytes, crown-like structures (CLS), are observed in large numbers in the male GWAT with very few in the male IWAT after HFD exposure. In contrast, female GWAT shows very few pro-inflammatory macrophages with HFD [23–25]. To assess sex differences in aging induced ATM induction we studied ATM profiles in the stromal vascular fraction (SVF) of GWAT and IWAT from young (6 months) and old (10 months) male and female mice that were placed on ND or HFD for 24-week (see Materials and Methods). Figure 2A shows the flow cytometry gating strategy to identify ATMs in young and old mice of both sexes. Initially, we performed immunofluorescence studies of ATMs in GWAT that showed obesity in older male GWAT led to the appearance of CLSs depicted by mac-2 staining for macrophages and cav-1 for adipocytes (Figure 2B; *top*). CLS were also depicted by H&E staining (Figure 2B; *bottom*) to be numerous and clustered closer together in males compared to females.

Given that both GWAT and IWAT expand with changes in diet and age, we examined both depots especially since ATMs are differential in number in subcutaneous and visceral fat depots. Consistent with prior studies in young mice [26] a robust ATM expansion was observed significantly in the GWAT than the IWAT with HFD. Obesity led to a drastic increase in ATM numbers in both young and old male GWAT, but with more subtle increases in female mice exposed to HFD (Figure 2C). In the IWAT, ATMs were not significantly different between young and old mice in the same diet groups, but older males had more IWAT ATMs (Supplementary Figure 1A). As expected in the GWAT, there were more CD11c^+^ATMs in the young obese males than females. Aging led to an increase in CD11c^+^ATMs in obese males but females showed no significant changes in the GWAT (Figure 2D). Comparatively obesity in young males produced more CD11c^+^ATMs than old obese males in GWAT and IWAT (Figure 2D and Supplementary Figure 1B). In contrast, aging led to an increase in GWAT CD11c^-^ATMs in lean and obese males compared to young males (Figure 2E). Females showed persistent predominance of CD11c^-^ATMs in young and old animals that increased with obesity in both GWAT and IWAT (Figure 2E and Supplementary Figure 1C).

These studies show that females regardless of age have attenuated inflammation with lower pro-inflammatory CD11c^+^ATMs but increased CD11c^-^ATMs with HFD. Overall, aging males appear to have more CD11c^-^ ATMs than young males. However, with obesity, young males show increased pro-inflammatory CD11c^+^ATMs, while aging males led to increased anti-inflammatory CD11c^-^ATMs.

### Lipolytic responses are enhanced in older obese females with ADRB3 stimulation

**Figure 3:**
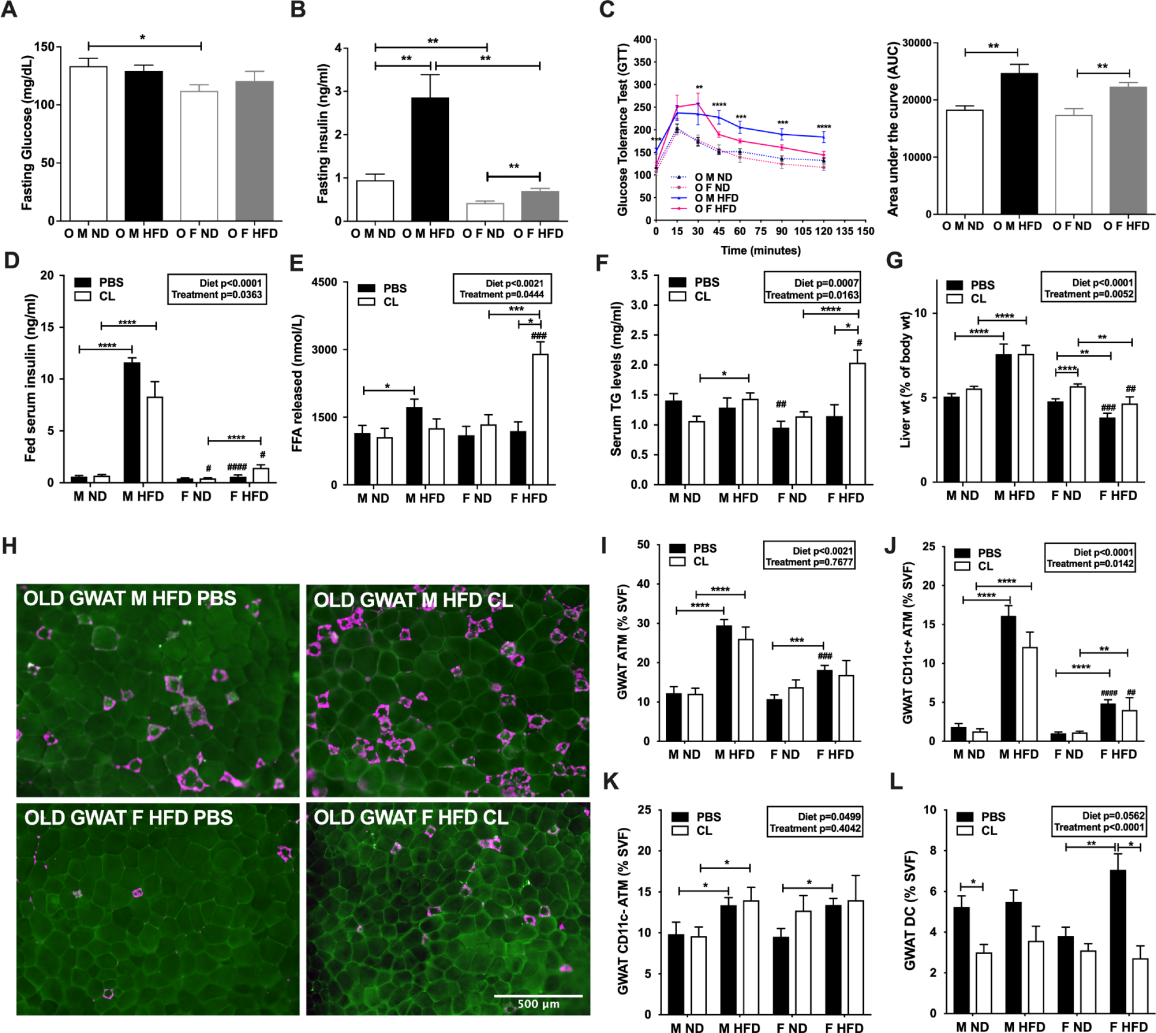
**ADRB3 stimulated lipolysis elevates lipolytic levels in old and obese females but does not affect CD11c ATM numbers in GWAT.** (**A**) Fasting glucose levels and (**B**) Fasting insulin levels at 10 weeks of HFD. (**C**) *left panel* - Glucose tolerance test (GTT) in 18-month-old male and female mice at 12 weeks of HFD; *right panel* - Area under the curve (AUC) from GTT. (**D**) Fed serum insulin levels in male and female obese mice after CL-316,243 (CL) treatment. (**E**) Serum FFA levels. (**F**) Serum TG levels. (**G**) Liver percent weight. (**H**) Immunofluorescence images of PBS or CL treated old male obese GWAT (*top row*) and old female obese GWAT (*bottom row*) depicting MAC-2 labeling (magenta) and CAV-1 labeling of adipocytes (green). Scale bar = 500 μm. Flow cytometry analysis and quantitation as a % of SVF of (**I**) GWAT ATMs (**J**) GWAT CD11c^+^ ATMs (**K**) GWAT CD11c^-^ ATMs (**L**) GWAT dendritic cells (DC) numbers. N=6-14 /group. One-way ANOVA with Student’s t-test was performed for (**A**–**C**). Two-way ANOVA with Bonferroni-Dunn’s post-test was performed for (**D**–**G**) and (**I**–**L**). Statistics from diet and sex interaction are in box. Statistical significance is indicated by *p <0.05, **p<0.01, ***p<0.001, ****p<0.0001. Student’s t-test was performed for male and female comparisons between the same diet groups indicated by ^#^p<0.05, ^##^p<0.01 ^###^p<0.001 and ^####^p<0.0001; error bars are SEM.

Fasting glucose levels at 10 weeks of HFD were comparatively lower in the old lean females than males (Figure 3A). Fasting insulin levels were determined to understand the impact of HFD in both sexes. Male mice on ND and HFD showed higher insulin levels than females (Figure 3B). Glucose tolerance testing (GTT) studies at 12 weeks of HFD showed that glucose tolerance was impaired in both older males and females on HFD, which is different than what we have observed in young females fed HFD [24] (Figure 3C). We have previously shown that stimulated lipolytic responses are sexually dimorphic in obese young mice [25, 26]. To determine whether there are sex differences in stimulated lipolysis in aging mice, we used the ADRB3 agonist; CL-316, 243 (CL) in old male and female C57Bl6/j mice placed on HFD at 10 months of age for 16- week (60% HFD) to induce obesity over the same length of time as our young mice experiments [24, 25, 27]. To determine the effects of lipolysis stimulation, fed serum insulin levels were determined with and without CL treatment. Fed serum insulin levels varied by diet and treatment in both sexes with older males on HFD showing higher insulin levels, while females showing significantly lower insulin levels than males in HFD condition with and without CL treatment (Figure 3D).

Serum FFA levels were assessed to determine overall lipolytic response. Both diet and CL treatment showed significant effects on the lipolytic response. Older males on HFD showed significantly greater lipolytic responses than males on ND (Figure 3E). Older obese female mice showed increased serum FFA responses compared to controls (Figure 3E). However, ADRB3 stimulation did not affect serum FFA levels in older obese male mice (Figure 3E). Serum TG estimation also showed significantly higher lipid levels in older obese females upon ADRB3 stimulation (^#^p<0.05, Figure 3F). Serum basal TG levels were also significantly lower in the older lean females than lean males (^##^p< 0.01, Figure 3F). No changes in body and adipose tissue weights were observed in either sex with or without HFD upon ADRB3 stimulation (data not shown). Circulating lipids are however often stored in the liver hence we next evaluated liver mass which varied by diet and treatment between sexes. Livers of older male mice on HFD with and without ADRB3 stimulation were significantly larger in weight than females (^##^p<0.01, ^###^p<0.001, Figure 3G), similar to results with 24-week of HFD (Figure 1D). Livers of older males with or without ADRB3 stimulation did not show any weight changes (Figure 3G). However, in control mice on ND, ADRB3 stimulation increased liver weights by percentage in females (Figure 3G).

To determine whether lipolytic responses might be a consequence of changes in sex steroid levels in aging, we determined estradiol and testosterone levels in young and old mice on ND and HFD in both sexes. Unexpectedly, only diet but not age had a significant effect on serum estradiol and testosterone levels (two-way ANOVA, p<0.05; Supplementary Figure 2A and 2B). Estrogen and testosterone levels remained unchanged in older obese females compared to young females (Supplementary Figure 2A and 2B). HFD exposure led to increased estrogen levels in young female mice compared to ND mice (Supplementary Figure 2A). HFD exposure also led to increased testosterone levels in older male mice compared to ND mice (Supplementary Figure 2B). These observations suggest that HFD exposure might affect sex steroid level production in females as well as males possibly due to changes in adipose tissue fat accumulation.

Overall, these results show that adipose tissue expansion in older female mice is prone to exaggerated lipolytic responses with ADBR3 agonist stimulation due to increased TG accumulation. Interestingly, despite lipolytic stimulation, the livers of older females showed decreased liver mass compared to males suggesting effective lipid clearance through a potentially high oxidative metabolism.

### ADRB3 stimulation failed to expand CD11c^-^ ATM accumulation in aged male and female GWAT

Previous studies from our lab and others show that FFAs are potential ligands for inflammatory pathways suggesting a link between lipolysis induced FFAs and inflammation [25, 28]. We previously identified that CL treatment proliferates CD11c^-^ ATMs in both males and females but to a greater extent in females [25]. To determine the effects of excess FFAs in circulation through ADRB3 stimulation in aged mice, we performed flow cytometry analyses of GWAT SVF from CL treated and untreated old ND and HFD male and female mice. CLS representative of ATMs were observed to be persistent in older male GWAT with and without CL treatment (Figure 3H, *top*). CLS were considerably fewer in the female old GWAT. ADRB3 stimulation with CL did not show any drastic increase in CLS in older female GWAT (Figure 3H, *bottom*).

Flow cytometry of GWAT SVF showed that diet had a significant effect on the induction of CD64^+^ ATMs in both older male and female GWAT, but lipolytic stimulation did not affect the overall ATM numbers (Figure 3I). In the lean state, older female GWAT had similar ATM numbers compared to males. However, older females on HFD had significantly fewer numbers of CD64^+^ ATMs than male GWAT (Figure 3I). Specifically, diet and CL treatment had a significant effect on CD11c^+^ ATMs, in the SVF, which were significantly lower in the old obese female GWAT compared to their male counterparts (Figure 3J) and remained unchanged with CL treatment. In the IWAT SVF as well, ATMs were significantly higher in both old males and females on a HFD (Supplementary Figure 2C). As expected, old and obese males showed a high density of CD11c^+^ and CD11c^-^ ATMs in IWAT compared to females (Supplementary Figure 2D and 2E). Interestingly, both the sexes equally generated CD11c^-^ ATMs in the GWAT and IWAT on HFD (Figure 3K and Supplementary Figure 2E). While we have previously observed that lipolysis induces CD11c^-^ ATMs in young obese mice [26], but in old mice ADRB3 stimulation failed to elicit any increase in CD11c^+^ or CD11c^-^ ATM response in both sexes in either fat depots (Figure 3J and 3K and Supplementary Figure 2D and 2E). In our previous study, dendritic cells (DCs) were observed to be reduced in young male and female obese GWAT and IWAT with stimulated lipolysis. In aged mice, ADRB3 stimulation reduced DC populations in lean male and obese female GWAT (Figure 3L) but such a response was not observed in the IWAT (Supplementary Figure 2F).

### GWAT and liver inflammatory responses to stimulated lipolysis are attenuated in older animals

**Figure 4:**
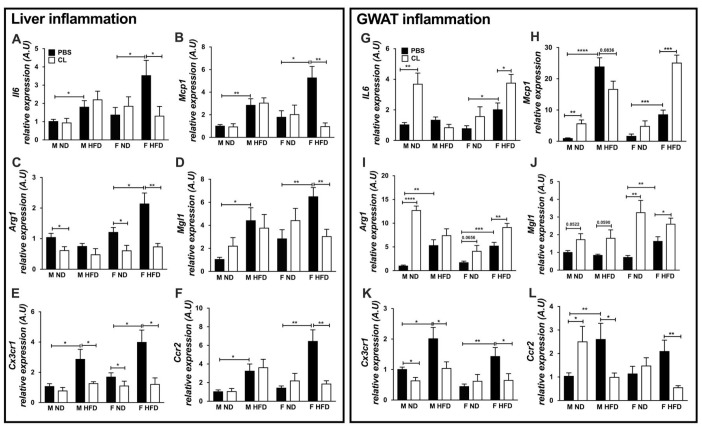
**ADRB3 stimulation promotes inflammatory cytokine and chemokine expression in old and obese female GWAT.** Expression of liver inflammation genes - (**A**) *Il6* (**B**) *Mcp1* (**C**) *Arg1* (**D**) *Mgl1* (**E**) *Cx3cr1* (**F**) *Ccr2.* Expression of GWAT inflammation genes - (**G**) *Il6* (**H**) *Mcp1* (**I**) *Arg1* (**J**) *Mgl1* (**K**) *Cx3cr1* (**L**) *Ccr2* in lean and obese male and female GWAT with and without ADRB3 stimulation. A.U., arbitrary units normalized to *Gapdh*, N=5-8. One-way ANOVA with Student’s t-test was performed for (**A**–**L**). Statistical significance is indicated by *p<0.05, **p<0.01, ***p<0.001, ****p<0.0001; error bars are SEM.

Studies have shown that aging increases the susceptibility of hepatic inflammation with HFD [26]. We hypothesized that liver inflammation in addition to inflammation in the visceral fat may contribute to metabolic impairment in older animals. To determine this, we investigated sex differences in the age-related increase in mRNA levels of classic markers of inflammation in adipose tissue and liver. We found that livers from old obese mice had higher mRNA expression of the cytokine; *Il6* and chemokine; *Mcp1* in both males and females (Figure 4A and 4B). CL treatment showed an effect only on *Mcp1* being reduced in the livers of the older females on HFD (Figure 4B). Hepatic *Arg1* and *Mgl1* (markers of regulatory macrophages) were both significantly higher in old females on HFD but in older obese males only *Mgl1* showed an increased expression (Figure 4C and 4D). ADRB3 stimulation showed a decrease in expression of *Arg1* and *Mgl1* in older obese females, while *Arg1* was also decreased in expression in older lean female livers (Figure 4C and 4D). We also determined chemokine receptor gene expression to assess recruitment of macrophages into the liver. Both *Cx3cr1* and *Ccr2* chemokine receptors were upregulated in the older male and female mice liver with HFD (Figure 4E and 4F). *Cx3cr1* expression was reduced upon ADRB3 stimulation both in the lean and obese states in females but in males in the obese state only (Figure 4E). *Ccr2* expression in the liver was reduced significantly in the older obese females upon ADRB3 stimulation (Figure 4F). Overall, this suggests that in obese older animals both regulatory and inflammatory monocytes are being recruited to the liver in response to dietary challenge.

Since GWAT is the primary site for inflammation in the HFD state, we determined the expression of inflammatory markers in the GWAT as well. GWAT cytokine markers *Il6* and chemokine *Mcp1* were significantly increased with HFD and ADRB3 stimulation in the old obese females (Figure 4G and 4H). In older male GWAT, ADRB3 stimulation increased *Il6* and *Mcp1* only in the lean state (Figure 4G and 4H). *Arg1* expression was elevated in lean male GWAT only with ADRB3 stimulation but showed significant effects in both the lean and older female GWAT upon ADRB3 activation (Figure 4I). On the other hand, *Mgl1* expression was observed to be increased with ADRB3 stimulation in both lean and obese male and female GWAT (Figure 4J). Overall, this suggested that in aged males on HFD, lipolysis did not enhance the existing inflammatory tone. HFD led to increased expression of chemokine receptor genes, *Cx3cr1* and *Ccr2* in the old male and female GWAT (Figure 4K and 4L). However, *Cx3cr1* and *Ccr2* were found to be significantly decreased with ADRB3 activation in the male and female obese GWAT (Figure 4K and 4L). Interestingly, in the old lean male GWAT, *Cx3cr1* mRNA expression was decreased with ADRB3 stimulation but *Ccr2* was elevated (Figure 4K and 4L). These results demonstrate that HFD-fed older females have a more robust anti-inflammatory response to lipolysis in GWAT with ADRB3 stimulation.

To determine differences in lipolysis and lipid metabolism due to aging and HFD, we also assessed markers responsive to lipolytic stimulation in the liver and GWAT. Lipolysis responsive genes such as *Atf3*, *Dio2* were significantly higher in expression upon CL treatment in older female GWAT with ND and HFD suggesting increased ADRB3 stimulation (Supplementary Figure 3A and 3B). However, the same was not observed in older obese male GWAT. *Ucp1* expression was also lower in older obese males than females (Supplementary Figure 3C). In old male GWAT, the mRNA levels of *Pparγ* and *Pgc1α* involved in oxidative metabolism were downregulated with obesity and with ADRB3 stimulation (Supplementary Figure 3D and 3E). Interestingly, in old female GWAT, the mRNA levels of *Pparγ* and *Pgc1α* were upregulated with obesity but downregulated with ADRB3 stimulation (Supplementary Figure 3D and 3E). Lipogenic gene expression levels of *Fasn*, and *Acsl* were significantly repressed with HFD in old female GWAT compared to males. However, ADRB3 stimulation decreased *Fasn* and *Acsl* expression in both old male and female GWAT (Supplementary Figure 3F and 3G). In contrast, in the liver, obesity led to higher expression of *Pparα* and *Pparδ* expression in older female livers compared to young females while ADRB3 stimulation led to downregulation of *Pparα* and *Pparδ* expression (Supplementary Figure 4A and 4B). Similar results were observed for lipogenic marker genes - *Fasn*, and *Acsl* suggesting a regulation of lipid accumulation, particularly in the older female livers (Supplementary Figure 4C and 4D). It is likely that even with obesity in aging, the persistent oxidative metabolism but repressed lipogenesis in older female GWAT and liver is a protective mechanism attributed to peripheral estrogen levels that keeps a check on inflammation.

### Senescence and fibrosis combined with greater fatty acid oxidation promote adipose tissue remodeling in old and obese female GWAT

**Figure 5:**
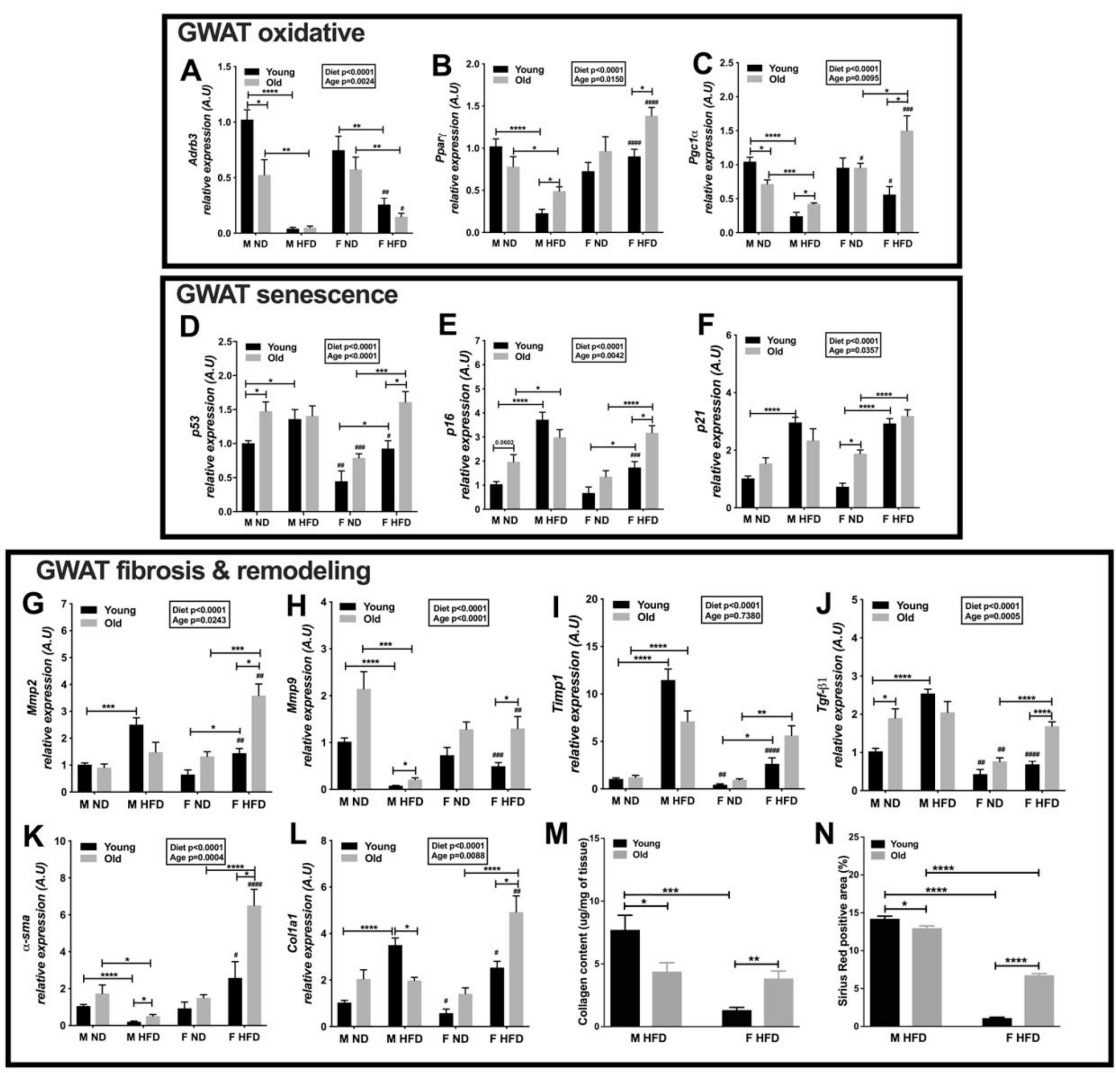
**Oxidative metabolism and ECM remodeling is highly active in older obese female mice.** Relative expression of GWAT oxidative genes - (**A**) *Adrb3* (**B**) *Pparγ* (**C**) *Pgc1α.* Expression of GWAT senescence genes (**D**) *p53* (**E**) *p16* (**F**) *p21.* Expression of GWAT fibrosis and ECM remodeling genes - (**G**) *Mmp2* (**H**) *Mmp9* (**I**) *Timp1* (**J**) *Tgfβ1* (**K**) *α-sma* (**L**) *Col1a1* in young and old obese male and female GWAT. A.U., arbitrary units normalized to *Gapdh*. N=5-8. (**M**) Collagen content in GWAT of young and old obese male and female mice, N=5. (**N**) Picrosirius red staining of young and old, male and female HFD GWAT positive area as a percentage. N=5. Two-way ANOVA with Bonferroni-Dunn’s post-test was performed for (**A**–**L**). Statistics from diet and sex interaction are in box. Statistical significance is indicated by *p<0.05, **p<0.01, ***p<0.001, ****p<0.0001. Student’s t-test was performed for male and female comparisons between the same diet groups indicated by ^#^p<0.05, ^##^p<0.01 ^###^p<0.001 and ^####^p<0.0001; error bars are SEM. One-way ANOVA with Student’s t-test was performed for (**M**–**N**). Statistical significance is indicated by *p<0.05, **p<0.01, ***p<0.001, ****p<0.0001; error bars are SEM.

To determine whether aging affects fatty acid oxidation and metabolism in the adipose tissue, we examined the mRNA levels of oxidative genes in the young and old male and female GWAT. Activation of ADRB3 receptors increased the oxidative capacity of tissues [29]. Both old age and HFD decreased the expression of *Adrb3* in male and female GWAT. However, in the HFD model, younger and older female GWAT expressed more *Adrb3* than the male GWAT (Figure 5A). The activity of nuclear receptors is important in lipid metabolism, for example *Pparγ* and *Pparδ* are linked to the maintenance of an alternative M2 activation state of CD11c^-^ ATMs in male mice [30, 31]. We examined whether changes in GWAT oxidative potential are associated with age related differences in *Pparγ* and *Pgc1α* expression. *Pparγ* and *Pgc1α* expression were found to be significantly associated with diet and age (Figure 5B and 5C). *Pparγ* and *Pgc1α* were expressed significantly more in the older obese male and female GWAT than the young mice (Figure 5B and 5C). Comparatively, their expression was much higher in the young and older female GWAT suggesting greater fatty acid oxidation in female GWAT than males (Figure 5B and 5C).

Alterations that occur in the adipose tissue compartment during obesity and aging can disrupt the adipocyte interaction with the extracellular matrix (ECM), as well as alter soluble and ECM-bound factors, thereby impairing stem cell homeostasis and promoting cell senescence [14]. Senescent markers such as *p53*, *p16* and *p21* are upregulated in aging and obesity [32, 33]. Therefore, we determined the changes in senescent markers in old and young GWAT of males and females. Expression of *p53*, *p16* and *p21* was significantly affected by diet and age (Figure 5D–5F). *p53* and *p16* expression were elevated in older lean male GWAT compared to young male GWAT (Figure 5D and 5E). On the other hand, *p21* expression was found to be higher in older lean females than young mice (Figure 5F). *p53*, *p16* and *p21* showed significantly higher expression in older lean males but lower expression in female GWAT (Figure 5D–5F). Obesity in young mice increased *p53, p16* and *p21* expression in male mice more than female GWAT (Figure 5D–5F). These results suggest that with aging and HFD, both males and females show signs of increased senescence in the GWAT that may affect the metabolic health of GWAT.

Whole-body metabolic homeostasis also depends on how adipose tissue remodels in response to excess calorie intake [32]. Matrix metalloproteinases (MMPs) and their inhibitors (tissue inhibitors of metalloproteinases; TIMPs) are the main actors in the ECM remodeling, and their behavior is altered in pathological states [34, 35]. *Mmp2* and *Mmp9* expression in the GWAT were significantly affected by diet and age (Figure 5G and 5H*). Mmp2* and *Mmp9* expression were higher in older obese female GWAT (Figure 5G and 5H), while *Mmp9* was highly expressed in older lean male GWAT (Figure 5H). The pro-fibrotic marker *Timp1* did not show any significant effects with age but diet showed a significant effect with the expression being higher in GWAT of both young and old male and female mice on HFD (Figure 5I). Overall, female GWAT had lower *Timp1* expression than males in the young mice (Figure 5I). Another fibrotic marker, *Tgf-β1* was found to be expressed significantly in lean older male GWAT and obese older females (Figure 5J). Overall *Tgf-β1* expression was lower in the young and old female GWAT (Figure 5J).

*α-sma* and *Col1a1* are *Tgf-β1* response genes involved in cytoskeletal changes necessary for efficient matrix contraction coupled with matrix tensile strength*. α-sma* is potentially important for the contractile features of cells under normal tissue repair and fibrotic conditions [36]. Collagens form one of the main families of ECM proteins, thereby playing a critical role in maintaining the structure of the majority of tissues [37]. Aging and obesity are characterized by collagen deposition in the adipose tissue [38]. The expression of *α-sma* and *Col1a1* in young and old GWAT were assessed, wherein both diet and age had a significant effect (Figure 5K and 5L). *α-sma* expression was found to be significantly higher in both older male and female GWAT on HFD (Figure 5K). Young lean and obese females had lower *Col1a1* expression than males (Figure 5L). On a HFD, older male GWAT showed lower expression, while older female GWAT showed higher expression of *Col1a1* (Figure 5L). To determine the extent of tissue fibrosis, we also determined the collagen content of GWAT in the young and old male and females on HFD. Young obese female GWAT had lower collagen content than young males. Interestingly, older obese female GWAT showed greater collagen content than young female GWAT (Figure 5M). On the other hand, younger male GWAT on HFD showed significantly increased collagen content than older obese male GWAT (Figure 5M). We also performed picrosirius red staining to visualize and determine fibrotic changes due to collagen deposition with aging and HFD in the GWAT of both sexes (Supplementary Figure 5). The percentage of fibrosis depicted by Sirius red positively stained area was significantly greater in the GWAT of young males compared to females on HFD (Figure 5N). While obesity in aging females led to greater fibrosis than young females, older obese male GWAT showed decreased fibrosis than young obese male GWAT (Figure 5N). The high expression of ECM and fibrotic markers suggest that the old obese female adipose tissue is metabolically active and pro-fibrotic to accommodate ECM structural changes.

These results demonstrate a potential for greater fatty acid oxidation along with an expression of markers involved in tissue remodeling in the obese females compared to males. Furthermore, a balance between oxidative metabolism and ECM remodeling in older female GWAT compared to the male counterparts would allow for greater GWAT adipocyte expansion.

## DISCUSSION

Studies comparing sex and age as factors mediating metabolism and metabolic inflammation are sparse in the literature. Men are prone to inflammation while younger women tend to be protected against metabolic risks likely due to ovarian hormones like estrogen [39–41]. However, post-menopausal aging women are more likely to be obese and at increased metabolic risk for diabetes and cardiovascular diseases [18, 19] making it imperative to assess the effects of aging in the female sex. We therefore investigated the sex differences in inflammatory, fibrotic and lipid metabolic responses in aging and obese male and female GWAT fat depot. We also studied whether increased FFA levels with stimulated lipolysis might generate a varied response in aged obese male and female mice. We identified that aging equalized female body weights similar to male mice in both lean and HFD challenged states. However, even with aging, females remained protected from obesity induced proinflammatory macrophages and stimulated lipolysis did not adversely affect the inflammatory state. Males continued to have more pro-inflammatory CD11c^+^ ATMs and higher insulin levels even though females had more visceral adiposity. Interestingly, obesity in aging demonstrated similar expression of senescent – *p53, p16* and *p21* and fibrotic markers -*Timp1* and *Tgf-β1* between male and female GWAT*.* However, adipogenic and oxidative genes such as *Pparγ,* and *Pgc1α* and ECM remodeling markers such as *Col1a1, α-sma, Mmp2* and *Mmp9* were upregulated in female GWAT suggesting improved tissue adaptability and metabolic health than males.

Within the visceral adipose tissue during aging, inflammation is linked to a systemic increase in pro-inflammatory cytokines in males [9]. In young mice, sex differences exist in fat storage, adipocyte remodeling, lipolytic and inflammatory responses [20, 26, 40]. In our studies with young and old mice on a 24-week HFD, in GWAT we observed sex differences in fat deposition in the visceral and subcutaneous fat depots. Old females showed an increased tendency to expand and store fat in GWAT and IWAT fat depots than old males. Alterations due to the HFD and the impact of age on inflammation were also reflected in the liver. Between the young and old males, lipid accumulation was also greater in the older male livers. A previous study in old male mice showed that aging increases the susceptibility of hepatic inflammation in response to HFD with marked infiltration of hepatic macrophages, and increased expression of inflammatory cytokines [26]. We observed significant expression of hepatic inflammatory markers, *Il6*, *Mcp1*, *Cx3cr1* and *Ccr2* in our studies (Figure 4) that might be a potential source for raising the GWAT inflammation in old obese males. Interestingly, older females also showed increased expression of these hepatic inflammatory markers but with lower lipid accumulation and with no changes in GWAT CD11c^+^ ATMs leading us to speculate that there might be sex differences in liver lipid metabolism.

The metabolic status of adipocytes is a major determinant of macrophage inflammatory response. In the young mice, as expected males showed large numbers of GWAT CD11c^+^ ATMs, while females had increased number of anti-inflammatory CD11c^-^ ATMs. This sex difference is dramatic in the visceral fat, but also holds true for subcutaneous IWAT. Aging influences the inflammatory profile and is associated with an increase in T cells and macrophages in the visceral fat depot in male mice [42]. With the fat extensively expanded in the older females, we expected to observe a rise in the inflammatory status in obese old females compared to the younger female mice. However, like young obese females, older obese females also showed persistent GWAT CD11c^-^ ATMs accumulation but no increases in CD11c^+^ ATMs. This could be attributed to protective effects of persistent estrogen levels in these older females with no significant increases in testosterone levels as observed in our studies.

Sex hormones play a critical role in body fat distribution and studies have shown a putative, protective role of estrogens in improving insulin resistance and dampening inflammation in obesity [21, 43, 44]. Hormonal changes in aging leads to redistribution of body fat to non-adipose tissues such as liver, skeletal muscle, heart, and pancreatic β-cells [13]. Models of estrogen deficiency such as ovariectomized [44] and aromatase knock out mice [45] also show increased visceral fat accumulation with greater inflammation implicating the role of sex hormones in depicting the inflammatory tone [46]. However, ovariectomy has been shown to accelerate aging and maybe a model for premature aging [47]. The ovariectomy model lacks sex steroids while the aging model depicts a gradual decline in sex hormones that closely resembles human biological aging in older men and postmenopausal women. Obesity induced metabolic status also led to changes in sex steroid production with an impact on inflammation. There was notably higher circulating estrogen levels in aging obese females with increased adiposity that might keep a check on their pro-inflammatory status. Contrary to a mouse model inhabited in regulated conditions, in humans, other stressors may play a part in increasing disease risk such as maternal programming of offspring obesity by HFD exposure and childhood obesity [48, 49]. Also, it has been observed that a group of metabolically healthy obese post-menopausal women presented a favorable inflammation profile despite increased adiposity [50]. There is a gap in some of the clinical literature with using age specific data or menstrual cycle length often in studies as opposed to direct measures of androgen and estrogen levels. However, it has been observed that women with a higher testosterone to estrogen ratio, in PCOS, do have a more similar phenotype (adipose inflammation and metabolism) to the older males in our studies [51, 52].

Although the old obese males in these studies continued to produce more CD11c^+^ ATMs compared to females, there was also an equal increase in CD11c^-^ ATMs as well. A possible reason for this could be declining testosterone levels associated with aging males that impact the inflammatory status in the obese male GWAT. In contrast, testosterone assessment in our studies showed no significant changes in the older males as well as females. Hence a limitation of our aging model was the inability to mimic hormonal modulations as in post-menopausal female and older male human subjects. Such discrepancies in the aging mouse model might be a consequence of sex steroid production by peripheral tissues. Previous studies have shown that adipose tissue is a primary peripheral source for estrogen production in both males and females [46, 53]. Despite low circulating levels in aging, local estrogen concentrations may be high, modifying the inflammatory status in both males and females. A future mice study with prolonged aging might be beneficial to recapitulate the human aging model. Another limitation of our study is the lack of assessment of other immune cells such as T and B cells. Ahnstedt et al showed that middle-aged females have pro-inflammatory T cells and decreased anti-inflammatory Tregs in adipose tissue [54]. Therefore, it is possible that obesity in aging females may lead to greater induction of T cells in obese female GWAT modifying the overall inflammatory status which remains to be studied.

We previously observed that obese young female mice showed fewer ATMs in GWAT, but adrenergic stimulation of lipolysis promoted CD11c^-^ ATM proliferation in GWAT [26]. We therefore hypothesized a change in the inflammatory profile with stimulated lipolysis in the aged mice. One limitation of our lipolysis study is the lack of lipolytic measures at early timepoints to better understand the initial inflammatory response to adrenergic stimulation and insulin action. In our previous study we showed that higher lipolytic levels in young obese female GWAT were attributed to increased pHSL levels compared to males [25]. However, we did not further investigate lipolytic proteins in our aging model. We speculate that higher lipolytic levels in older obese females might be a consequence of increased pHSL and higher estrogen levels. Lipolytic stimulation enhanced expression of GWAT *Il6* and *Mcp1* but also led to an increased expression of anti-inflammatory genes *Arg1* and *Mgl1* in aging obese females that might provide protection against CD11c^+^ pro-inflammatory ATMs in GWAT. In contrast, old obese males showed no ATM changes with ADRB3 stimulation and no inflammatory response to the lipolysis possibly due to the prior presence of a higher inflammatory tone in obese old male GWAT fat pads. A result of this inflammatory tone in male GWAT was demonstrated by enhanced fibrosis in the tissues as seen in immunofluorescence imaging demonstrating clustering of CLSs. Inflammatory cytokine and chemokines in aging could be a trigger for ECM modulation through fibrosis and senescence [55]. The presence of CD11c^-^ ATMs and a greater fat storage/expansion capacity in old female GWAT led us to investigate the degree of fatty acid oxidation. Although *Adrb3* expression was lower with HFD and old age in both male and female GWAT, females showed significantly greater expression that might explain the higher lipolytic levels observed in females than males. This also led to aging obese females having relatively higher expression of *Pparγ* and *Pgc1α* suggesting an efficient fatty acid oxidative metabolism than males. Changes in fat metabolism would potentially lead the ECM being dynamically modified to meet the needs of the adipose tissue environment. Additional studies to further understand oxidative metabolism such as *ex vivo* explant studies with adrenergic stimulation or *in vivo* metabolic measurements might be beneficial in a future study.

Maintenance of a high degree of flexibility in the ECM network with fat expansion is a prerequisite for healthy adipose tissue. Adipose tissue expansion occurs dynamically through adipocyte hypertrophy and/or hyperplasia and requires continuous ECM remodeling to accommodate this expansion [32]. In our studies with young and old mice, aging led to upregulation of fibrotic markers – *Timp1* and *Tgf-β1* in the old obese female GWAT along with ECM remodeling markers - *Mmp2, Mmp9, Col1a1* and *α-sma.* Studies have shown that the matrix itself may promote differentiation of progenitor cells into adipocytes to accommodate further fat storage [32, 56, 57]. Increased MMP-2 and MMP-9 enzyme expression during adipocyte differentiation may promote adipogenesis and correlates with expression of proinflammatory markers [35]. Overall, this suggests that in aged female adipose tissue the fibrotic response to adipose expansion with HFD is geared towards a more adipogenic and regulatory environment.

In order to further understand differences in flexibility of adipocytes in aging obese females we turned to evaluations of senescence in both sexes. Unexpectedly we found that both obese males and females showed similar upregulation of senescent markers- *p53*, *p16* and *p21*. It is still possible that there are intrinsic sex differences in the ECM responses as studies in humans show that sex-specific changes in type I, III and IV collagen turnover was present at the age around menopause (age 40–60) with women having an increased turnover than males [58]. A limitation of our study is that we were unable to ascertain the direct involvement of ECM environment on adipose tissue metabolism through gene expression studies. We believe that higher expression of ECM components might not necessarily define the tissue as fibrotic in a pathological sense but may be a marker of remodeling that is transiently occurring with changes in metabolism. It is likely that the reduced ability to degrade and remodel the ECM during aging and obesity may exert a barrier against healthy adipose tissue expansion ECM remodeling in males. However, with further aging in females, the GWAT may lose its ability to expand and renew similar to older males. In this context, further studies are required to assess causality of ECM deposition on adipose dysfunction.

In summary, our studies demonstrate that aging and obesity in aging induce sexually dimorphic inflammatory responses owing to altered lipid metabolism and adipose tissue remodeling even with the same HFD exposure. Since older female adipose depots have enhanced lipid storage, it cannot be ruled out that over time females may eventually develop greater lipotoxicity resulting in increased susceptibility to secondary health issues that increase in incidence with aging, such as cardiovascular disease and ischemic stroke. Overall, the results of our study highlight the importance of using aged animals of both sexes in obesity-related investigations and emphasize the need for sex-based intervention strategies in older individuals.

## MATERIALS AND METHODS

### Animals and CL-316, 243 treatment

Young mice used in the experiments were male and female C57Bl/6J purchased from Jackson Laboratories. Old mice were either aged from mice purchased from Jackson or obtained from the National Institute of Aging (NIA). Mice were fed *ad libitum* either a control ND consisting of 13% fat (5001, Lab Diet) or a HFD of 60% of calories from fat (D12492, Research Diets, Inc.) starting at 6-week of age (young) or 10 months of age (old) for 16 to 24-week duration. Animals were housed in a specific pathogen-free facility with a 12h light/12h dark cycle and given free access to food and water. All animal use was in compliance with the Institute of Laboratory Animal Research Guide for the Care and Use of Laboratory Animals and approved by the University Committee on Use and Care of Animals at the University of Michigan (Animal welfare assurance number A3114-01. For stimulation of lipolysis, mice were injected intraperitoneally with 1 mg/kg of CL-316, 243 (CL; Tocris Bioscience), an ADRB3 selective agonist or saline twice (4 h between each injection). Mice were sacrificed 14 h after the second injection [25, 59]. Liver, GWAT and IWAT were excised and weighed for each animal. Tissue samples were frozen in liquid nitrogen and stored at –80°C prior to RNA extraction. Adipose tissues were collected and immediately processed for SVF preparation and flow cytometry staining.

### Immunohistochemistry and Immunofluorescence staining

Antibodies used for immunofluorescence included polyclonal anti-caveolin (CAV-1; Cell Signaling) and anti-Mac2 (MAC-2; Galectin-3; eBioM3/38; eBioscience). For histology, tissues were formalin fixed, paraffin embedded, sectioned at 5μm and stained with hematoxylin and eosin. Hematoxylin-eosin (H-E) staining was performed by the University of Michigan’s Comprehensive Cancer Center Histology Core.

### SVF isolation and flow cytometry

Adipose tissue fractionation and flow cytometry analysis were performed as described previously. SVF cells were stained with CD64 PE, CD45.2 e450, and CD11c-APC-Cy7 (eBioscience), and gating was performed for macrophage populations and by CD45 gates to determine ATMs [60, 61].

### Quantitative real-time PCR

RNA was extracted from adipose tissue and liver using Trizol LS (Life Technologies) and cDNA was generated using a High-Capacity cDNA Reverse Transcription Kit (Applied Biosystems). SYBR Green PCR Master Mix (Applied Biosystems) and the StepOnePlus System (Applied Biosystems) were used for real-time quantitative PCR. *Gapdh* expression was used as an internal control for data normalization. Samples were assayed in duplicate, and relative expression was determined using the 2^-ΔΔ^ CT method. All primers used are listed in Supplementary Table 1.

### Serum estradiol and testosterone estimation

Estradiol and testosterone levels were assessed in fed serum samples in young and old, male and female mice on 16 weeks of ND or HFD. Testing was performed at the Ligand Assay and Analysis Core Laboratory (University of Virginia).

### Serum insulin, fatty acid, glycerol and triglyceride estimation

Serum insulin levels were determined with an ultrasensitive insulin ELISA kit (Crystal Chem). For *in vivo* lipolysis measurement, serum FFA was measured using the HR NEFA series (Wako Diagnostics). Serum free glycerol was measured using free glycerol determination reagent (Sigma-Aldrich). Serum TG was measured with Infinity triglyceride determination kit (Thermo Scientific) following manufacturer’s instructions. Liver triglycerides were homogenized, extracted by a modified Folch method and assayed.

### Picrosirius red staining and quantification

Briefly, deparaffinized and rehydrated GWAT sections were stained by dissolving Sirius Red F3B (Sigma-Aldrich) in a saturated aqueous solution of picric acid (Sigma-Aldrich) at 0.1% w/v for 60 minutes at room temperature. Thereafter, these slides were washed in acidified water containing 0.5% glacial acetic acid (Sigma-Aldrich) twice for 5 minutes. After which the excess water was wicked and sections dehydrated in 3 changes of 100% ethanol, cleared with xylene, and mounted with Permount (Fisher Scientific). Bright-field images of stained GWAT sections were quantified using the ImageJ software (NIH) as per the protocol described on its website [62].

### Collagen extraction and assay

100 mg of adipose tissue was homogenized in 0.5 M acetic acid. The homogenate was transferred to an eppendorf tube and incubated at 4°C overnight on a shaker to allow acid solubilization. Following which the homogenate was centrifuged at 10,000 rpm and the supernatant collected with a syringe to get rid of excess fat. Rat tail collagen was used a standard for the assay. For the assay, 1ml of sircoll dye (0.1% Direct Red 80 in saturated picric acid) was added to the samples and standards and incubated on mechanical shaker for 1h. thereafter tubes were centrifuged at 10,000 rpm for 15 mins. The unbound dye solution was aspirated leaving the pellet behind to which 1 ml of 0.5M NaOH was added, vortexed and incubated on a shaker for 30 mins to release the bound dye in solution. 100ul of the dissolved dye solution was transferred to a 96-well plate and the absorbance was measured at 540nm.

### Statistical analyses

All values are reported as the mean +SEM unless otherwise stated. Statistical analyses were performed in Prism 8 (GraphPad). Before performing the statistical test, we assumed equal variability of differences among the groups and also checked for outliers by ROUT’s test with Q= 1%. Depending on the dataset, ordinary one-way analysis of variance (ANOVA) with Sidak’s post-test or Student’s t-test was performed. Ordinary two-way analysis of variance (ANOVA) was performed with factors of diet and age/treatment with Bonferroni-Dunn’s post-test to determine significance. The test performed is indicated in the figure legend. Statistical significance is indicated by *p<0.05, **p<0.01, ***p<0.001, ****p<0.0001.

## Supplementary Material

Supplementary Figures

Supplementary Table 1
