# Supplementary material for: Female adipose tissue has improved adaptability and metabolic health compared to males in aged obesity
Source: Aging (Albany NY). 2020 Jan 26;12(2):1725–46. doi: 10.18632/aging.102709 (PMC7053605; doi:10.18632/aging.102709)
Supplement: Supplementary Figures [file aging-12-102709-s002..pdf]

SUPPLEMENTARY FIGURES

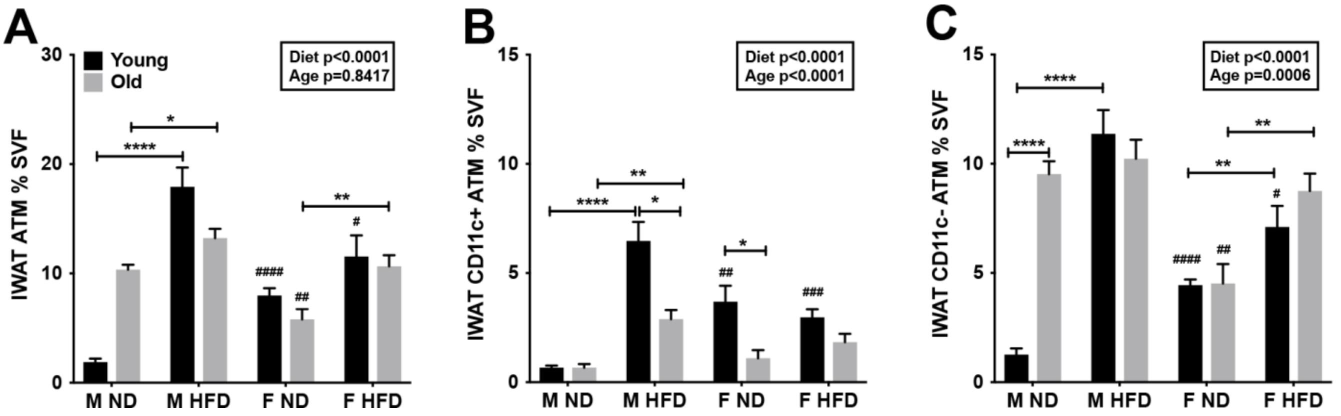

Supplementary Figure 1. Flow cytometry assessment of ATMs in young and old mice IWAT. Quantitation as a % of SVF of (**A**) IWAT ATMs (**B**) IWAT CD11c$^+$ ATMs (**C**) IWAT CD11c$^-$ ATMs in 24-week HFD fed young and old mice. N=7-12/group. Two-way analysis of variance (ANOVA) with Bonferroni-Dunn's post-test was performed for (**A**–**C**). Statistics from diet and sex interaction are in box. Statistical significance is indicated by *p<0.05, **p<0.01, ***p<0.001, ****p<0.0001. Student's t-test was performed for male and female comparisons between the same diet groups indicated by $^{\#}$p<0.05, $^{\#\#}$p<0.01 $^{\#\#\#}$p<0.001 and $^{\#\#\#\#}$p<0.0001; error bars are SEM.

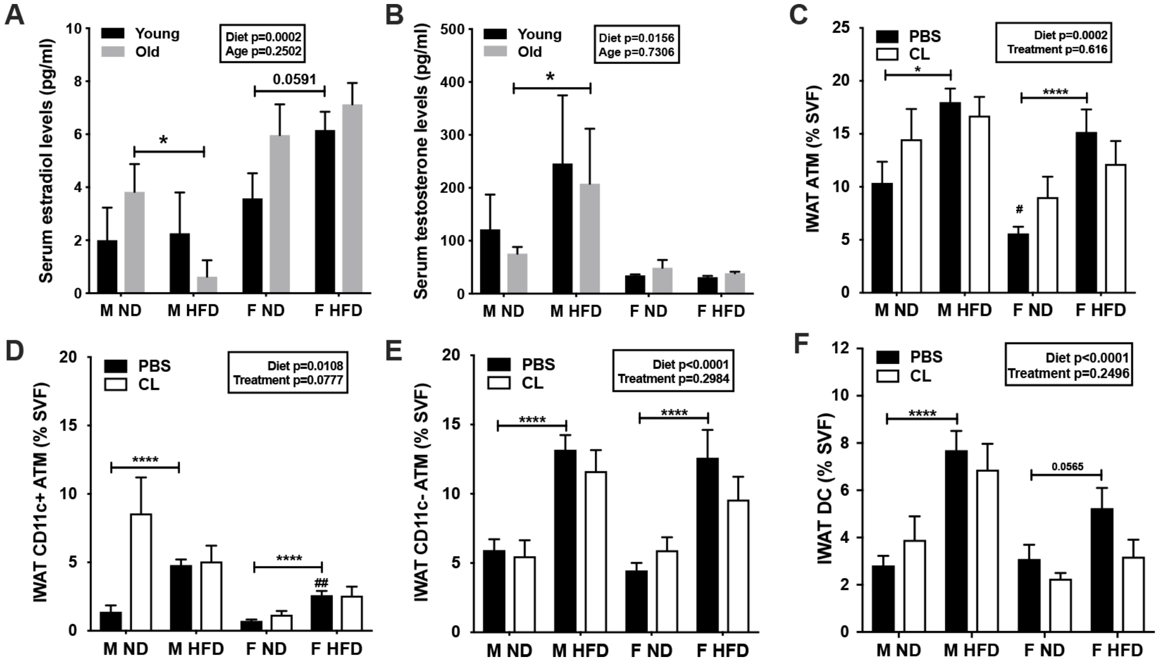

Supplementary Figure 2. Flow cytometry assessment of ATMs in old male and female mice IWAT with lipolytic stimulation. (**A**) Serum estradiol ELISA (**B**) Serum testosterone ELISA after 16 weeks of HFD. Quantitation as a % of SVF of (**C**) IWAT ATMs (**D**) IWAT CD11c$^+$ ATMs (**E**) IWAT CD11c$^-$ ATMs (**F**) IWAT dendritic cells (DC) numbers in 16-week HFD fed old mice treated with and without ADRB3 stimulation. N=6-14/group. Two-way analysis of variance (ANOVA) with Bonferroni-Dunn's post-test was performed for (**A**–**F**). Statistics from diet and sex interaction are in box. Statistical significance is indicated by *p<0.05, **p<0.01, ***p<0.001, ****p<0.0001. Student's t-test was performed for male and female comparisons between the same diet groups indicated by $^{\#}$p<0.05, $^{\#\#}$p<0.01 $^{\#\#\#}$p<0.001 and $^{\#\#\#\#}$p<0.0001; error bars are SEM.

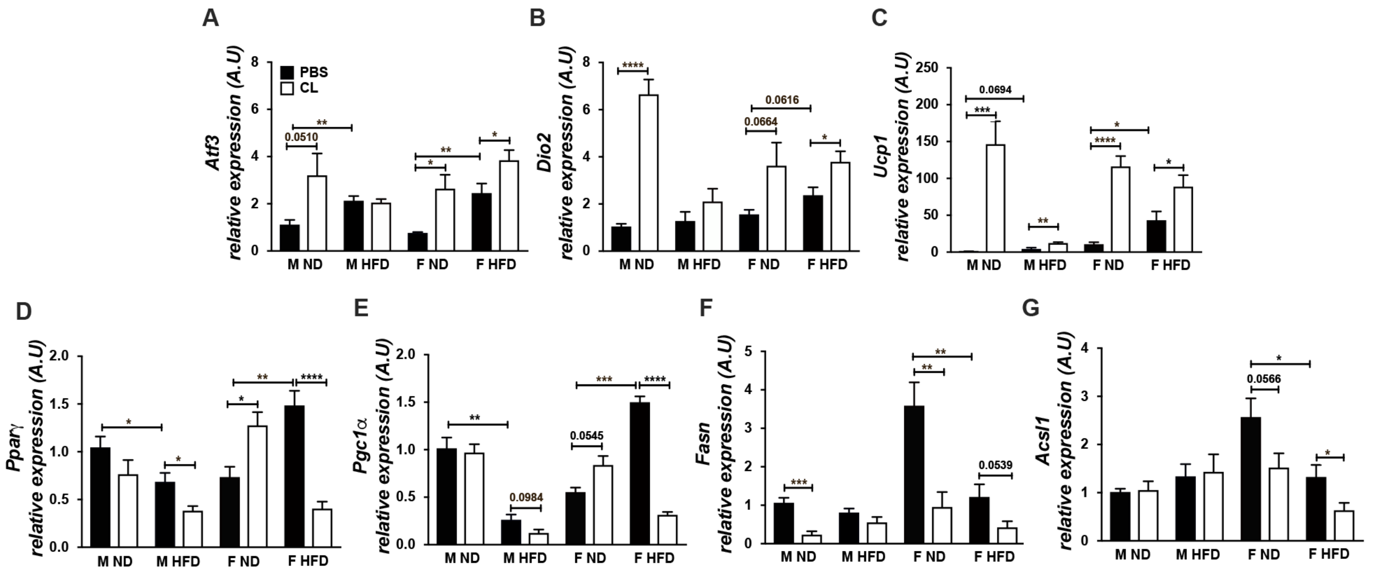

Supplementary Figure 3. Lipolysis responsive genes in old GWAT. Relative expression of GWAT (**A**) *Atf3* (**B**) *Dio2* (**C**) *Ucp1* (**D**) *Pparγ* (**E**) *Pgc1α* (**F**) *Fasn* (**G**) *Acsl1* in old obese male and female GWAT with and without CL treatment. A.U., arbitrary units normalized to Gapdh. N=5-8. One-way analysis of variance (ANOVA) with Student's t-test was performed for (**A**–**F**). Statistical significance is indicated by *p<0.05, **p<0.01, ***p<0.001, ****p<0.0001; error bars are SEM.

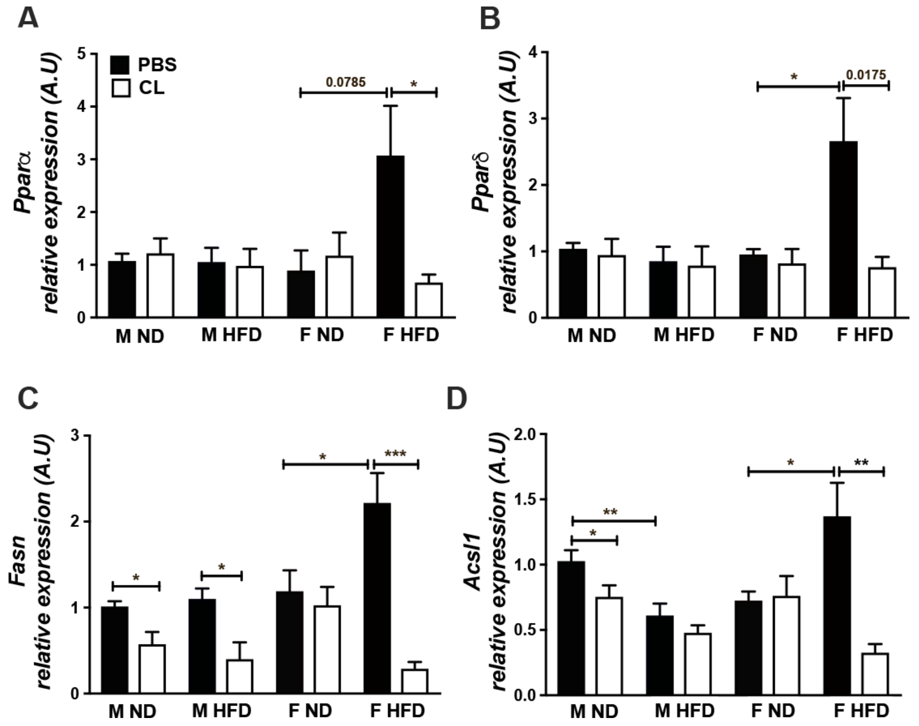

Supplementary Figure 4. Lipolysis responsive genes in old liver. Relative expression of GWAT (**A**) *Pparα* (**B**) *Pparδ* (**C**) *Fasn* (**D**) *Acsl1 in* old obese male and female liver with and without CL treatment. A.U., arbitrary units normalized to *Gapdh*. N=5-8. Oneway analysis of variance (ANOVA) with Student's t-test was performed for (**A**–**D**). Statistical significance is indicated by *p<0.05, **p<0.01, ***p<0.001, ****p<0.0001; error bars are SEM.

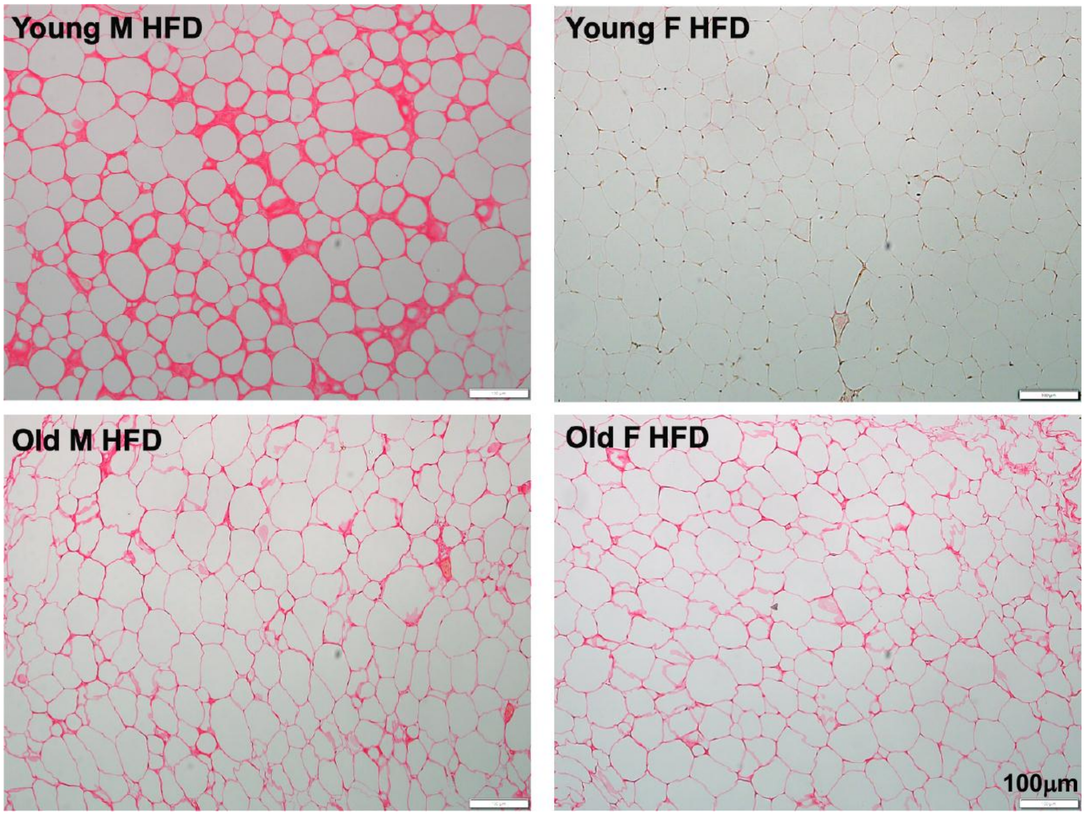

Supplementary Figure 5. Picro Sirius Red staining in young and old GWAT. GWAT sections showing Sirius red staining in *top row left* - Young male HFD; *top row right* - Young female HFD; *bottom row left* - Old male HFD; *bottom row right* - Old female HFD. Scale bar = 100 µm.
