# Supplementary material for: Female adipose tissue has improved adaptability and metabolic health compared to males in aged obesity
Source: Aging (Albany NY). 2020 Jan 26;12(2):1725–46. doi: 10.18632/aging.102709 (PMC7053605; doi:10.18632/aging.102709)
Supplement: Supplementary Table 1 [file aging-12-102709-s001..pdf]

## SUPPLEMENTARY TABLE

**Supplementary Table 1. Primers sequences for RT-PCR.**

| | Forward primer | Reverse primer |
|---|---|---|
| *Gapdh* | TGAAGCAGGCATCTGAGGG | CGAAGGTGGAAGAGTGGGAG |
| *Il6* | TAGTCCTTCCTACCCCAATTTCC | AAGGAACCCTTAGAGTGCTTACT |
| *Arg1* | CTCCAAGCCAAAGTCCTTAGAG | AGGAGCTGTCATTAGGGACATC |
| *Mcp1* | TTAAAAACCTGGATCGGAACCAA | GCATT AGCTT CAGATTT ACGGGT |
| *Mgl1* | TGAGAAAGGCTTTAAGAACTGGG | GACCACCT GT AGT GAT GT GGG |
| *Cx3cr1* | GCAAGCT CAC GACT GCCTTC | TCCGGTTGTTCATGGAGTTGG |
| *Ccr2* | ATCCACGGCATACTATCAACATC | CAAGGCTCACCATCATCGTAG |
| *Adrb3* | ACAGGAATGCCACTCCAATC | TTAGCCACAACGAACACTCG |
| *Pgcla* | TATGGAGTGACATAGAGTGTGCT | CCACTTCAATCCACCCAGAAAG |
| *Pparg* | GGAAGACCACT CGCATT CCTT | TCGCACTTTGGTATTCTTGGAG |
| *p16* | CATGTTGTTGAGGCTAGAGAGG | CACCGTAGTTGAGCAGAAGAG |
| *p53* | GCCATGGCCATCTACAAGAA | AATTTCCTTCCACCCGGATAAG |
| *p21* | AAGTGTGCCGTTGTCTCTTC | AGTCAAAGTTCCACCGTTCTC |
| *Timpl* | AGGTGGTCTCGTTGATTTCT | GTAAGGCCTGTAGCTGTGCC |
| *Tgf-b* | GT GGAAAT CAACGGGAT CAG | ACTTCCAACCCAGGTCCTTC |
| *Collal* | GCTCCTCTTAGGGGCCACT | CCACGTCTCACCATTGGGG |
| *a-sma* | GTTCAGTGGTGCCTCTGTCA | ACTGGGACGACATGGAAAAG |
| *Mmp2* | CAAGTTCCCCGGCGATGTC | TTCTGGTCAAGGTCACCTGTC |
| *Mmp9* | CACCACCACAACTGAACCAC | CTCAGAAGAGCCCGCAGTAG |
| *Atf3* | TGCCTGCAGAAAGAGTCAGAGA | AGCTCCTCGATCTGGGCC |
| *Dio2* | CCAGCACCGGAAAGAGGAAA | TCCTTGCACCATGACCCAAA |
| *Ppara* | AACATCGAGTGTCGAATATGTG | AGCCGAATAGTTCGCCGAAAG |
| *Fasn* | GGAGGTGGTGATAGCCGGTAT | TGGGTAATCCATAGAGCCCAG |
| *Ascll* | TGCCAGAGCTGATTGACATTC | GGCAT ACCAGAAGGT GGT GAG |
